# Inverted Internal Limiting Membrane Flap Technique versus Internal Limiting Membrane Peeling for Vitrectomy in Highly Myopic Eyes with Macular Hole-Induced Retinal Detachment: An Updated Meta-Analysis

**DOI:** 10.1155/2020/2374650

**Published:** 2020-08-24

**Authors:** Ling Ling, Yao Liu, Baixing Zhou, Feifei Gao, Zhe Hu, Man Tian, Yiqiao Xing, Kaibao Ji, Tao Sun, Wentian Zhou

**Affiliations:** ^1^Affiliated Eye Hospital of Nanchang University, Nanchang, Jiangxi, China; ^2^Department of Ophthalmology, Renmin Hospital of Wuhan University, Wuhan, Hubei, China

## Abstract

**Background:**

The aim of this meta-analysis was to compare morphological and functional outcomes between vitrectomy with the inverted internal limiting membrane (ILM) flap technique and vitrectomy with internal limiting membrane peeling in highly myopic eyes with macular hole- (MH-) induced retinal detachment (MHRD).

**Methods:**

The PubMed, Web of Science, Embase, and Cochrane Library databases were comprehensively searched from inception to November 10, 2019, for published studies comparing the two techniques for the treatment of MHRD. The outcomes in the collected articles included the postoperative MH closure rate, retinal reattachment rate, and best-corrected visual acuity (BCVA). Review Manager (version 5.3) was used for analyses.

**Results:**

In total, seven retrospective studies comparing the inverted ILM flap technique with ILM peeling for the treatment of MHRD were included. The MH closure rate was significantly higher in the inverted ILM flap group than in the ILM peeling group at 6 and 12 months after initial surgery (OR = 15.39; 95% CI: 6.68 to 35.43;*P* < 0.00001 and OR = 12.58, 95% CI: 3.51 to 45.08; *P*=0.0001), while the retinal reattachment rate was similar in both groups at 6 months after initial surgery (OR = 2.40; 95% CI: 0.89 to 6.50; *P*=0.08). Besides, the postoperative BCVA was significantly better in the inverted ILM flap group than in the ILM peeling group at 12 months after initial surgery (MD = −0.35; 95% CI: −0.52 to −0.18; *P* < 0.0001).

**Conclusions:**

Thus, the MH closure rate and postoperative BCVA may be better with the inverted ILM flap technique than with ILM peeling for myopic MHRD, while the postoperative retinal reattachment rate appears to be similar with both techniques. Therefore, in the future, vitrectomy with the inverted ILM flap technique should be preferred over standard ILM peeling technique for the treatment of MHRD in highly myopic eyes.

## 1. Introduction

Retinal detachment (RD) resulting from the macular hole (MH), also known as MH-induced RD (MHRD), most commonly occurs in eyes with high myopia and results in irreversible visual disorders [1]. The incidence of MHRD accounts for nearly 0.5%–5% of all cases of rhegmatogenous RD worldwide [2]. The potential pathogenesis may be related to tangential macular traction due to the premacular membrane and posterior vitreous cortex complex, inverse traction caused by a posterior staphyloma, or retinal pigment epithelium (RPE) atrophy [3, 4]. With an increase in the incidence of high myopia, the number of cases of MHRD has also increased dramatically. Therefore, treatment for MHRD associated with high myopia should be considered a public health problem.

Conventional approaches, including macular buckling, scleral shortening, and scleral imbrication, have been attempted to improve the surgical outcomes of eyes with MHRD [5–7]. With the introduction of pars plana vitrectomy (PPV) for the treatment of idiopathic MH, vitrectomy with removal of the cortical vitreous and internal limiting membrane (ILM) or gas tamponade has been widely accepted as the standard treatment for MHRD [8, 9]. Although ILM peeling for MHRD reportedly achieves a high retinal reattachment rate that ranges from 70% to 100% [10, 11], the MH closure rate is relatively low, ranging from 10% to 70% [12]. In eyes without MH closure after surgery, recovery of visual acuity is poor despite successful retinal reattachment [13]. In addition, studies have revealed that MH closure after PPV for MHRD in myopic eyes is an important prognostic factor for the recovery of visual acuity [14, 15].

Recently, Michalewska et al. proposed the inverted ILM flap technique for the treatment of large MHs [16] and myopic MHs with or without RD [17, 18]. This technique was found to enhance the postoperative MH closure rate and improve the postoperative visual acuity [16, 18]. However, it remains unclear whether the inverted ILM flap technique is a suitable procedure for the treatment of MHRD in highly myopic eyes. Although previous studies have compared the inverted ILM flap technique with the ILM peeling technique for MHRD, the results were controversial [19, 20]. Two previous meta-analyses also compared the two techniques for the treatment of MHRD; however, the results were contradictory in terms of the retinal reattachment rate and the follow-up periods were inadequate [21, 22]. Therefore, we performed an updated meta-analysis to compare the efficacy of vitrectomy with the inverted ILM flap technique with that of vitrectomy with ILM peeling for the treatment of MHRD in highly myopic eyes.

## 2. Materials and Methods

### 2.1. Search Strategy

This was a meta-analysis of previously published studies, so the need for ethical approval was waived. The meta-analysis was performed in accordance with the Preferred Reporting Items for Systematic Reviews and Meta-analysis guidelines [23]. Two reviewers (Ling Ling and Kaibao Ji) independently searched the PubMed, Web of Science, Embase, and Cochrane Library databases from inception to November 10, 2019, for all published papers comparing the inverted ILM flap technique with ILM peeling for MHRD. The search terms were as follows: (((high myopia) OR highly myopic)) AND (((((retinal perforations OR macular hole OR retinal break OR retinal tear OR retinal hole OR MH)) AND (Retinal detachment OR Retinal Pigment Epithelial Detachment OR RD)) AND (internal limiting membrane peeling OR ILM peeling OR internal limiting membrane removal OR inner limiting membrane peeling OR inverted internal limiting membrane flap technique OR inverted internal limiting membrane insertion OR internal limiting membrane repositioning OR ILM flap)) AND (Vitrectomy OR Vitrectomies)). Only manuscripts written in English were considered.

### 2.2. Inclusion and Exclusion Criteria

Studies were considered eligible if they met the following criteria: (1) original studies; (2) patients with high myopia (a spherical equivalent refractive error of at least −6.0 diopters (*D*) and an axial length of at least 26.5 mm) [24] and MHRD; (3) comparing outcomes of inverted ILM flap technique versus ILM technique for the treatment of myopic MHRD; (4) evaluation of the postoperative MH closure rate, retinal reattachment rate, and BCVA and the preoperative BCVA as primary outcomes; (5) postoperative follow-up evaluations at 6 and 12 months; and (6) availability of all primary outcome data for calculation of odds ratios (OR), mean differences (MDs), and 95% confidence intervals (CIs).

We excluded case reports, abstracts, posters, letters, reviews, meta-analyses, and surgical techniques; studies published in languages other than English; studies with insufficient data; studies that did not meet the inclusion criteria; studies where included eyes exhibited peripheral retinal breaks, proliferative vitreoretinopathy, idiopathic or traumatic MH, or proliferative diabetic retinopathy before surgery; and studies where the surgery also involved macular buckling.

### 2.3. Data Collection and Quality Assessment

Two authors (Ling Ling and Kaibao Ji) independently searched and extracted data from the included studies, and any disagreements were resolved through discussion. The following variables were collected from each included study: first author, publication year, study location, study design, number of eyes, mean age, sex ratio, axial length, outcomes, and follow-up duration. The methodological quality of case-control studies was assessed using the Newcastle-Ottawa Scale, which provides a score range of 0 to 9 points, with a higher score (≥5) indicating better quality [25].

### 2.4. Statistical Analysis

Review Manager software (Version 5.30, the Cochrane Collaboration, Oxford, England) was used to analyze the extracted data. The odd ratios (ORs) and their 95% confidence intervals (CIs) were used to estimate dichotomous variables. Mean differences (MDs) and their 95% confidence intervals (CIs) were used to calculate continuous variables. Chi-square statistic test and I^2^ statistic test were used to evaluate the heterogeneity among studies. I^2^ values of 25%, 50%, and 75% represented low, moderate, and high heterogeneity, respectively. If there was no significant heterogeneity among studies, a fixed-effect model was used. Otherwise, a random-effect model was employed. Funnel plots were used to assess the publication bias. *P* < 0.05 was considered significantly different among studies.

## 3. Results

### 3.1. Search Results

**Figure 1:**
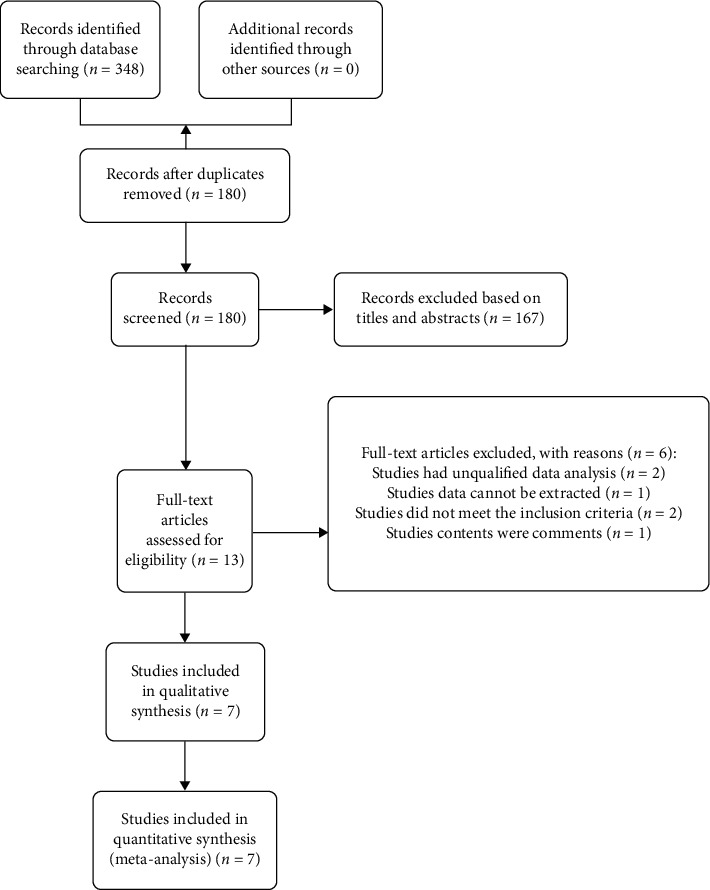
Flow diagram of the article selection process for meta-analysis.

The selecting process of studies is illustrated via a flow chart in Figure 1. A total of 348 potentially relevant studies were identified through our preliminary literature search (PubMed: 84, Web of Science: 122, Embase: 122, Cochrane Library: 20). From these, 168 studies were excluded because of duplication. Subsequently, 167 studies were excluded after screening of the titles and abstracts. The full text of the remaining 13 studies was evaluated; two studies had unqualified data, two did not meet the inclusion criteria, one was contextually irrelevant, and one contained data that could not be extracted. Eventually, seven studies [19, 20, 26–30] with a total of 198 eyes (84 in the inverted ILM flap group and 114 in the ILM peeling group) were included in our meta-analysis.

The characteristics of the seven studies are depicted in Table 1, and the results of their quality assessment are reported in Supplementary Table 1.

### 3.2. Meta-Analysis Results

**Figure 2:**
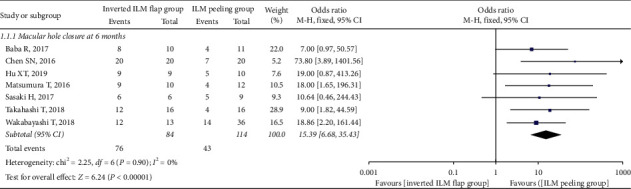
Forest plot comparing macular hole closure rate between inverted ILM flap and ILM peeling groups at 6 months after surgery. ILM = internal limiting membrane.

**Figure 3:**
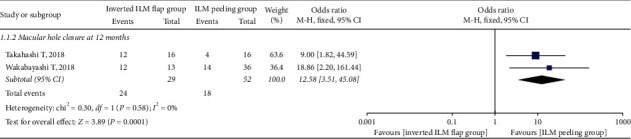
Forest plot comparing macular hole closure rate between inverted ILM flap and ILM peeling groups at 12 months after surgery. ILM = internal limiting membrane.

In total, 198 eyes, including 84 in the inverted ILM flap group and 114 in the ILM peeling group, exhibited MH closure at 6 months after initial surgery. The pooled OR for MH closure between the inverted ILM flap and ILM peeling groups was 15.39 (95% CI: 6.68 to 35.43; *P* < 0.00001; Figure 2) and with no heterogeneity (chi^2^ = 2.25; P = 0.90; I^2^ = 0%; Figure 2), indicating that the MH closure rate was higher in the inverted ILM flap group. We also calculated the MH closure rate at 12 months after initial surgery, and the result was in favor of the inverted ILM flap group (OR = 12.58; 95% CI: 3.51 to 45.08; *P*=0.0001; Figure 3), with no heterogeneity (chi^2^ = 0.30; P = 0.58; I^2^ = 0%; Figure 3).

**Figure 4:**
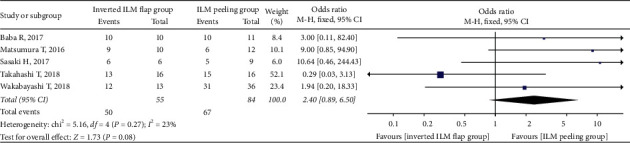
Forest plot comparing retinal reattachment rate between inverted ILM flap and ILM peeling groups at 6 months after surgery. ILM = internal limiting membrane.

The retinal reattachment rate showed no significant difference between the inverted ILM flap and ILM peeling groups at 6 months after surgery (OR = 2.40; 95% CI: 0.89 to 6.50; *P*=0.08; Figure 4), and the heterogeneity was low (chi^2^ = 5.16; *P*=0.27; I^2^ = 23%; Figure 4).

**Figure 5:**
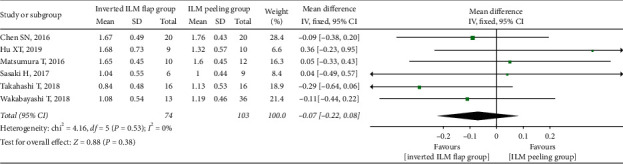
Forest plot comparing preoperative BCVA between inverted ILM flap and ILM peeling groups. BCVA = best-corrected visual acuity, ILM = internal limiting membrane.

**Figure 6:**
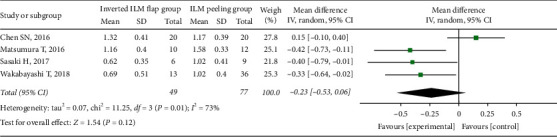
Forest plot comparing postoperative BCVA between inverted ILM flap and ILM peeling groups at 6 months after surgery. BCVA = best-corrected visual acuity, ILM = internal limiting membrane.

**Figure 7:**
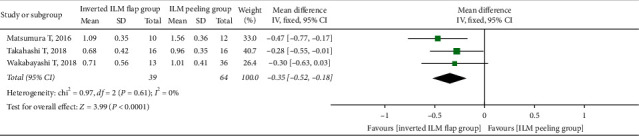
Forest plot comparing postoperative BCVA between inverted ILM flap and ILM peeling groups at 12 months after surgery. BCVA = best-corrected visual acuity, ILM = internal limiting membrane.

There was no significant difference in the preoperative BCVA between the two groups (MD = −0.07; 95% CI: −0.22 to 0.08; *P*=0.38; Figure 5), with no heterogeneity (chi^2^ = 4.16; *P*=0.53; I^2^ = 0%; Figure 5). We simultaneously pooled the postoperative BCVA data obtained at 6 and 12 months after surgery and found that the postoperative BCVA at 6 months was better in the inverted ILM flap group than in the ILM peeling group, although the difference was not significant (MD = −0.23; 95% CI: −0.53 to 0.06; *P*=0.12; Figure 6). However, the postoperative BCVA at 12 months was significantly better in the inverted ILM flap group than in the ILM peeling group (MD = −0.35; 95% CI: −0.52 to −0.18; *P* < 0.0001; Figure 7), with no heterogeneity (chi^2^ = 0.97; *P*=0.61; I^2^ = 0%; Figure 7).

In terms of macular structure, we analyzed the ellipsoid zone (EZ), revealing that the pooled OR in the inverted ILM flap group and the ILM peeling group at 6 months after surgery was comparable (OR = 4.49; 95% CI: 0.79 to 25.66; *P*=0.09; Supplementary Figure 1), with no heterogeneity (chi^2^ = 0.12; *P*=0.73; I^2^ = 0%; Supplementary Figure 1).

### 3.3. Publication Bias

**Figure 8:**
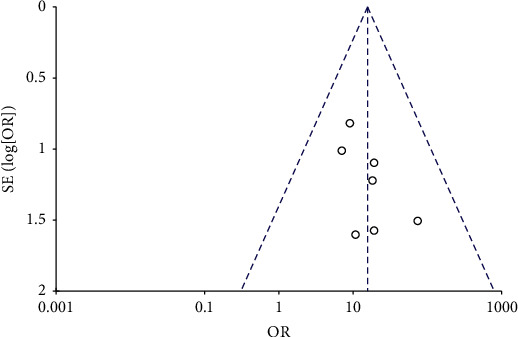
A funnel plot of MH closure rate at 6 months after surgery showing no significant publication bias. MH = macular hole, SE = standard error, OR = odds ratio.

**Figure 9:**
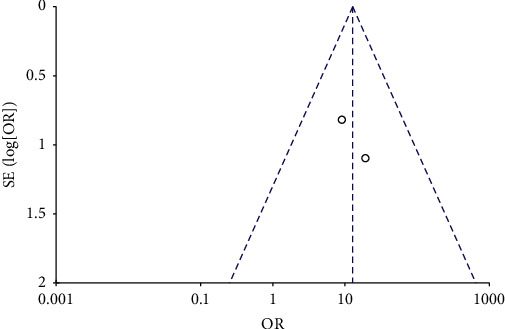
A funnel plot of MH closure rate at 12 months after surgery showing no significant publication bias. MH = macular hole, SE = standard error, OR = odds ratio.

**Figure 10:**
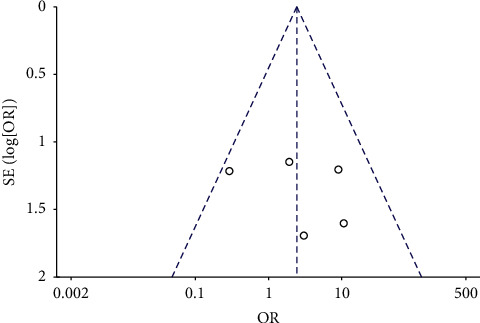
A funnel plot of retinal reattachment rate showing no significant publication bias. SE = standard error, OR = odds ratio.

**Figure 11:**
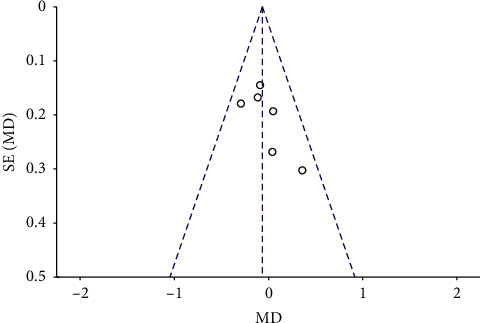
A funnel plot of preoperative BCVA showing no significant publication bias. BCVA = best-corrected visual acuity, SE = standard error, MD = mean difference.

**Figure 12:**
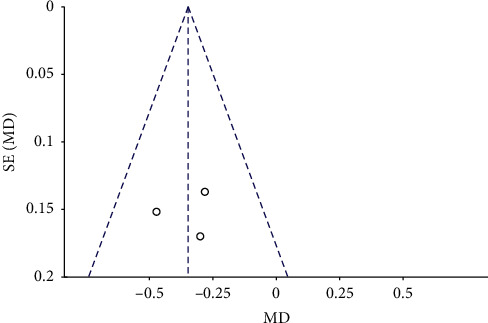
A funnel plot of postoperative BCVA at 12 months after surgery showing no significant publication bias. BCVA = best-corrected visual acuity, SE = standard error, MD = mean difference.

The funnel plots for the MH closure rate at 6 and12 months after surgery, retinal reattachment rate at 6 months after surgery, preoperative BCVA, postoperative BCVA at 12 months after surgery, and EZ were symmetrical, indicating no significant publication bias (Figures 8–12, Supplementary Figure 2).

## 4. Discussion

We analyzed seven eligible studies comparing vitrectomy with the inverted ILM flap technique and vitrectomy with ILM peeling for the treatment of MHRD in highly myopic eyes. The pooled results indicated that the MH closure rate and postoperative BCVA were significantly better with the former procedure, although the retinal reattachment rate and EZ were comparable between groups. In addition, the preoperative BCVA was similar in both groups. Compared with the conventional ILM peeling technique, the inverted ILM flap technique appears to improve not only the anatomical outcomes but also the functional results.

Internal limiting membrane peeling has become a widely performed maneuver in vitreoretinal surgery, being the treatment of choice for idiopathic macular hole [31]. While no proved efficacy has been shown for this additional step in epiretinal membrane surgery [32], ILM peeling provided encouraging outcomes when associated with retinal detachment surgery [33]. When it comes to MHRD in highly myopic eyes, ILM peeling ensured a high rate of primary reattachment and macular hole closure, compared to non-ILM peeling [34]. However, the management of MHRD in highly myopic eyes is challenging and several surgical approaches have been proposed, including the inverted ILM flap technique [17, 18].

The MH closure rate (OR = 12.58) was significantly higher in the inverted ILM flap group than in the ILM peeling group at 12 months after initial surgery. Michalewska proposed that an inverted ILM flap provides a scaffold for glial cells to proliferate and eventually promotes successful MH closure [16]. Another hypothesis is that proliferating glial cells may fill MH and compensate for the relative shortening of the retina [20]. A previous study also demonstrated a higher MH closure rate with the inverted ILM flap technique than with ILM peeling for MHRD in eyes with high myopia [18]. Therefore, the inverted ILM flap technique can improve the MH closure rate while reducing the risk of recurrent retinal detachment.

Although the retinal reattachment rate (OR = 2.40) in the inverted ILM flap group was higher than that in the ILM peeling group at 6 months after surgery, the difference was not significant. Moreover, the heterogeneity was low. This may be attributed to the study of Takahashi et al. [29], who reported a lower postoperative retinal reattachment rate for the inverted ILM flap group. This result accounted for a certain weight in our meta-analysis, leading to opposite statistical results. Another possible explanation is the relatively small sample size. Further studies with larger samples are needed to clarify this aspect.

With regard to the postoperative BCVA, although the value for the inverted ILM flap group was not significantly higher than that for the ILM peeling group at 6 months after surgery, the higher MH closure and retinal reattachment rates in the inverted ILM flap group indicated better visual acuity. The lack of a significant difference at 6 months may be associated with the lower mean BCVA in the study of Chen and Yang [26]. A potential factor contributing to this heterogeneity was the staining dye; while Chen et al. used indocyanine green (ICG) [26], the other three studies used brilliant blue G (BBG) [20, 28, 30]. A previous study also showed that ICG may have toxic effects on the retinal tissue, such as RPE atrophy [35]. However, there was a significant intergroup difference in the postoperative BCVA at 12 months after surgery, with no heterogeneity. This could be attributed to the use of BBG for ILM staining in all three included studies [28–30]. Glial cell proliferation induced by the inverted ILM flap, as proposed by Michalewska, produces an environment for photoreceptors to assume new positions in direct proximity to the fovea, thus resulting in improved postoperative visual acuity [16]. In one study, spectral domain optical coherence tomography (OCT) showed that the presence of the external limiting membrane (ELM) and ellipsoid zone (EZ) line in the foveal area was stronger in the inverted ILM flap group than in the ILM peeling group, and there was a positive correlation between a better BCVA and the presence of ELM and the EZ line [27]. We have also shown that EZ restoration was better in the inverted ILM flap group than that of ILM peeling group. Our result is similar to that of another study where the inverted ILM flap technique resulted in a significantly better postoperative BCVA than did the ILM peeling technique [30].

At present, the selection of surgical methods for MHRD is mostly based on the diameter of the hole. For small MH, less than 400 *μ*m, the closure rate was close to 90% with the use of ILM peeling surgery, which is associated with a low rate of recurrence and retinal detachment [36–38]. However, for large MH, more than 400 *μ*m, inverted ILM flap technique was performed for better closure rate and postoperative visual acuity [16, 39].

This study had certain limitations. First, the number of eyes was relatively small and the quality of evidence relatively low, probably because retrospective case-control studies were analyzed. Second, the follow-up period of 12 months was not adequate for the evaluation of long-term anatomical and functional outcomes for the macula.

Third, the analysis of the macular microstructure observed using OCT was not sufficient, and correlation between the macular structure and postoperative visual acuity was not evaluated. Fourth, the mean change in BCVA from the preoperative to the postoperative period and complications should be reported during the follow-up period. Finally, publication bias may have resulted in heterogeneity in terms of the postoperative BCVA. Further large-scale, prospective studies with longer follow-up periods are necessary to confirm our results.

In conclusion, the findings of this updated meta-analysis provide evidence for better MH closure rates and visual outcomes with the inverted ILM flap technique than with ILM peeling for myopic MHRD. The retinal reattachment rate seems to be similar with both techniques. Therefore, in the future, vitrectomy with the inverted ILM flap technique should be preferred over standard ILM peeling technique for the treatment of MHRD in highly myopic eyes.

## Figures and Tables

**Table 1:** The baseline patient characteristics of the included studies.

Study	Country	Study type	Number of participants (eyes)	Mean age (years)	Gender (female/male)	Axial length (mm)	Outcome measures	Follow-up
Baba et al. [19]	Japan	Retrospective observational study	ILM flap: 10	74 (55–89)	5/5	28.95(27.03–31.00)	MH closure, retinal reattachment, BCVA	9.4 (6.0–27.5)
			ILM Peeling:11	68 (55–88)	8/3	30.30(27.80–32.28)		19.4 (6.0–48.4)
Sasaki et al. [20]	Japan	Retrospective study	ILM flap: 6	75.0 ± 6.4	5/1	30.47 ± 2.57	MH closure, retinal reattachment, BCVA	6
			ILM peeling: 9	66.0 ± 12.5	7/2	30.10 ± 1.95		6
Chen and Yang [26]	Taiwan	Retrospective interventional study	ILM flap: 20	62.06 ± 8.90	4/16	28.40 ± 1.94	MH closure, BCVA	8.92 ± 3.23
			ILM peeling: 20	60.53 ± 8.78	6/14	29.35 ± 1.88		14.13 ± 6.66
Hu et al. [27]	China	Retrospective study	ILM flap: 19	58.8 ± 13.8	16/3	29.4 ± 1.9	MH closure, BCVA	17.6 ± 11.3
			ILM peeling: 21	59.9 ± 8.5	15/6	30.0 ± 2.4		23.4 ± 16.1
Matsumura et al. [28]	Japan	Retrospective interventional study	ILM flap: 10	67.7 ± 9.7	8/2	28.4 ± 2.2	MH closure, retinal reattachment, BCVA	12
			ILM peeling: 12	75.3 ± 8.7	11/1	30.4 ± 1.6		12
Takahashi et al. [29]	Japan	Retrospective study	ILM flap: 16	68.4 ± 7.8	14/2	29.1 ± 1.9	MH closure, retinal reattachment, BCVA	12
			ILM peeling: 16	69.1 ± 8.5	15/1	29.6 ± 1.1		12
Wakabayashi et al. [30]	Japan	Retrospective study	ILM flap: 13	67.8 ± 9.9	11/2	29.4 ± 0.9	MH closure, retinal reattachment, BCVA	12
			ILM peeling: 36	69.2 ± 9.1	34/2	29.6 ± 1.7		12

## Supplementary Materials

Supplementary Materials Supplementary Table 1: NOS for assessing quality of the included studies. Supplementary Figure 1: Forest plot comparing postoperative EZ restoration between inverted ILM flap and ILM peeling groups at 6 months after surgery. Supplementary Figure 2: a funnel plot of postoperative EZ restoration showing no significant publication bias.
