# Supplementary material for: Inverted Internal Limiting Membrane Flap Technique versus Internal Limiting Membrane Peeling for Vitrectomy in Highly Myopic Eyes with Macular Hole-Induced Retinal Detachment: An Updated Meta-Analysis
Source: J Ophthalmol. 2020 Aug 24;2020:2374650. doi: 10.1155/2020/2374650 (PMC7463360; doi:10.1155/2020/2374650)
Supplement: Supplementary Materials — Supplementary Table 1: NOS for assessing quality of the included studies. Supplementary Figure 1: Forest plot comparing postoperative EZ restoration between inverted ILM flap and ILM peeling groups at 6 months after surgery. Supplementary Figure 2: a funnel plot of postoperative EZ restoration showing no significant publication bias. [file 2374650.f1.docx]

| Methodological item for non-randomized  studies (No.1- 8) | Baba R, 2017[19] | Sasaki H, 2017[20] | Chen SN, 2016[26] | Hu XT, 2019[27] | Matsumura  T, 2016^[28]^ | Takahashi  H, 2018^[29]^ | Wakabayashi  T, 2018^[30]^ |
| --- | --- | --- | --- | --- | --- | --- | --- |
| 1. Is the Case Definition Adequate? | 1 | 1 | 1 | 1 | 1 | 1 | 1 |
| 2. Representativeness of the Cases | 1 | 1 | 1 | 1 | 1 | 1 | 1 |
| 3. Selection of Controls | 0 | 0 | 0 | 0 | 0 | 0 | 0 |
| 4. Definition of Controls | 0 | 0 | 0 | 0 | 0 | 0 | 0 |
| 5. Comparability of Cases and Controls on  the Basis of the Design or Analysis | 2 | 2 | 2 | 2 | 2 | 2 | 2 |
| 6. Ascertainment of Exposure | 1 | 1 | 1 | 1 | 1 | 1 | 1 |
| 7.Same method of ascertainment for cases  and controls | 1 | 1 | 1 | 1 | 1 | 1 | 1 |
| 8. Non-Response Rate | 1 | 1 | 1 | 1 | 1 | 1 | 1 |
| Total score | 7 | 7 | 7 | 7 | 7 | 7 | 7 |

Supplementary Table 1. NOS for assessing quality of the included studies.

NOS= Newcastle-Ottawa Scale; The selection area included Nos. 1–4, which was up to one score in one question; The comparability area included No. 5, which was up to 2 scores in the question; The exposure area included Nos. 6–8, which was up to one score in one question. The total score was 9.

Supplementary Figure 1：Forest plot comparing postoperative EZ restoration between Inverted ILM flap and ILM peeling groups at 6 months after surgery.


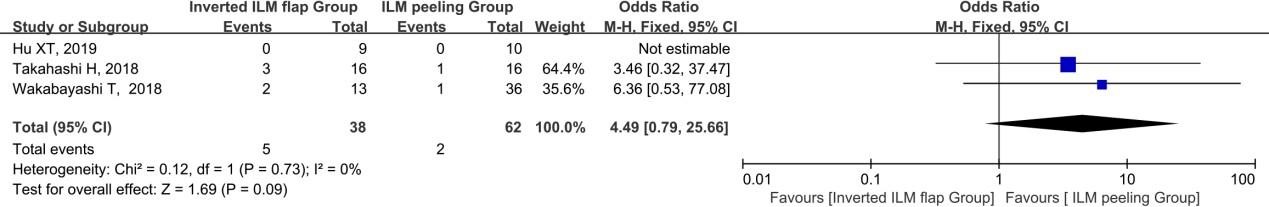
EZ = ellipsoid zone; ILM= internal limiting membrane

Supplementary Figure 2：A funnel plot of postoperative EZ restoration showing no significant publication bias. EZ = ellipsoid zone; ILM= internal limiting

membrane


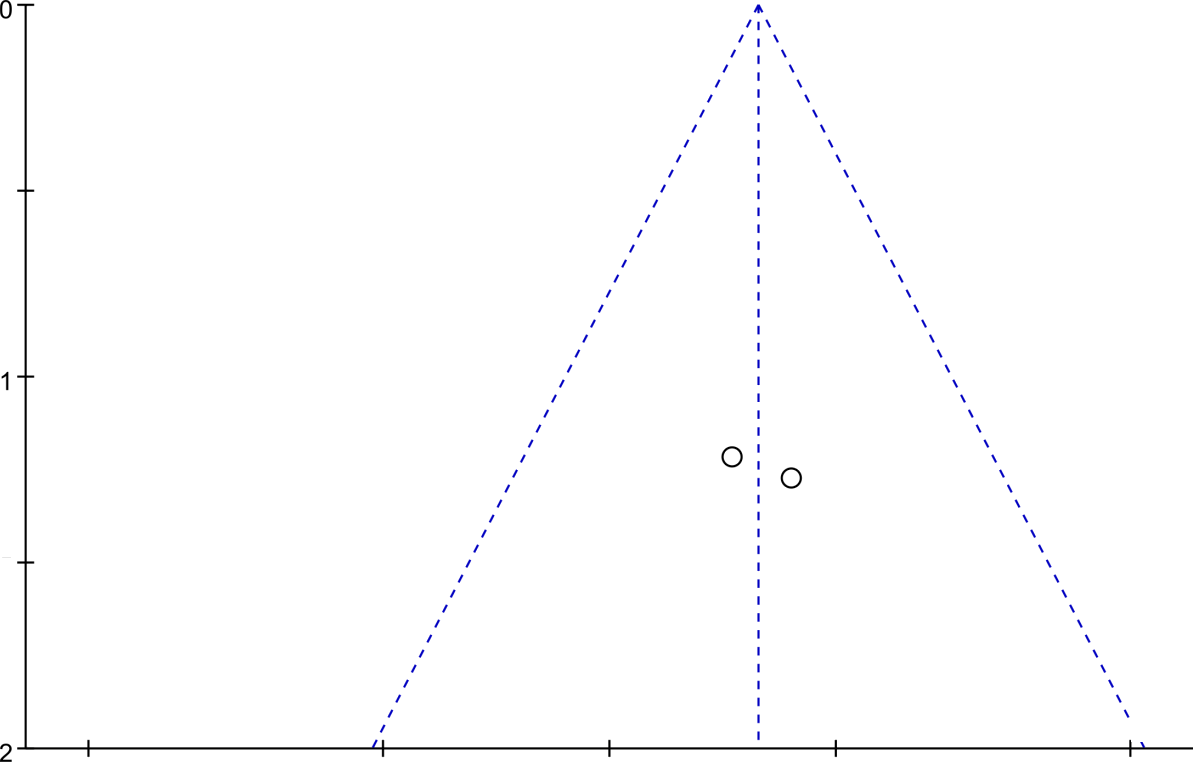

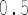

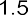

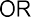


0.005 0.1 1 10
